# Adaptability of the Soybean Aphid *Aphis glycines* (Hemiptera: Aphididae) to Temperature and Photoperiod in a Laboratory Experiment

**DOI:** 10.3390/insects15100816

**Published:** 2024-10-17

**Authors:** Bo Gao, Kaice Yang, Yifan Tian, Bing Bai, Zhenqi Tian, Jian Liu

**Affiliations:** 1 College of Plant Protection, Northeast Agricultural University, Harbin 150030, China; 13045148976@163.com (B.G.); yangkaice2023@163.com (K.Y.); a3156711162@163.com (Y.T.); 18346552530@163.com (B.B.); tianzq@neau.edu.cn (Z.T.); 2 Graduate School of Chinese Academy of Agricultural Sciences, Chinese Academy of Agricultural Sciences, Beijing 100081, China; 3 Key Laboratory of Economic and Applied Entomology of Liaoning Province, College of Plant Protection, Shenyang Agricultural University, Shenyang 110866, China

**Keywords:** *Aphis glycines*, wild soybean, morph differentiation, temperature adaptability, photoperiod adaptability

## Abstract

The soybean aphid, *Aphis glycines* Matsumura, 1917, is a crucial soybean pest. Cultivated soybean, *Glycine max* (Carl von Linné) Elmer Drew Merrill, 1917, and wild soybean, *Glycine soja* Philipp Franz von Siebold & Joseph Gerhard Zuccarini, 1843, are summer hosts of *A. glycines*. In this study, the development, reproduction, and morphogenesis of *A. glycines* fed wild soybean (*Ag*FW) were studied at different temperatures and photoperiods. The data were compared with that of *A. glycines* fed soybean (*Ag*FS). At 20–29 °C, the adult lifespan of the first–third-generation *Ag*FW was shorter than or equal to that of *Ag*FS. Significant differences existed in the adult fecundity and intrinsic rate of increase between *Ag*FW and *Ag*FS. At a 10L:14D h photoperiod, males of *Ag*FW were deposited earlier than, or as early as, males of *Ag*FS. At 17 °C, the gynoparae of *Ag*FW were deposited in proportions greater than or equal to those of *Ag*FS. Based on these results, we concluded that the adaptability of *Ag*FW and *Ag*FS to temperature and photoperiod significantly differs. It is important to understand the life cycle of *A. glycines* in Harbin, northeast China, and formulate an integrated pest management strategy for *A. glycines* in the region.

## Simple Summary

The soybean aphid, *Aphis glycines*, is a crucial soybean pest, which has two major hosts, cultivated and wild soybean. This study mainly proved that the *A*. *glycines* fed on wild soybean exhibited significant differences in morph differentiation, temperature adaptability, and photoperiod adaptability, compared with the *A*. *glycines* fed on cultivated soybean. It is important to understand the life cycle of *A. glycines* in Harbin, northeast China, and formulate an integrated pest management strategy for *A. glycines* in the region.

## 1. Introduction

The soybean aphid, *Aphis glycines* Matsumura, 1917, is a crucial soybean pest native to Asia. *Aphis glycines* is a species with an expanding range, which is acquiring new territories, including North America. After 2000, *A. glycines* invaded North America and spread rapidly throughout the region [1]. The life cycle of *A. glycines* is heteroecious and holocyclic (host alternating with sexual reproduction during part of its life cycle) [2,3]. In summer, *A. glycines* virginoparae colonize cultivated soybean, *Glycine max* (Carl von Linné) Elmer Drew Merrill, 1917, and reproduce parthenogenetically throughout the season [4,5]. As the temperature decreases and the photoperiod shortens in autumn, gynoparae, males, and oviparae of *A. glycines* are deposited [1,6]. Soybeans infested with *A. glycines* display leaf shrinkage, internode shortening, plant dwarfing, and reduced pod number [7]. Through direct feeding [2] and indirect damage by virus transmission [8,9], *A. glycines* has caused up to 30% yield loss in soybeans during certain years with heavy infestations [7].

The morphological characteristics, population dynamics, and natural enemies of *A. glycines* have been studied in various regions [10,11,12,13,14,15,16]. The economic threshold used in *A. glycines* management is 250 aphids per plant [17,18]. In the control of this pest, insecticides [19,20,21], seed treatment [22], and gene silencing [23] have been applied. The natural control of *A. glycines* was mainly resistant plants, natural enemy insects, and microorganisms, which were reported in previous studies [5,15,16]. The adaptability of *A. glycines* to constant day–night and fluctuating temperatures has been studied [24,25,26,27]. At day–night constant temperatures ranging from 13 to 33 °C, *A. glycines* nymphs developed into adults [28]. At a constant 35 °C, *A. glycines* nymphs did not develop into adults, and all died within 8 d [29]. The population growth rates of *A. glycines* were greatest at 25 °C, with the aphid population doubling in size in 1.5 days [25]. At diurnal 35 °C and nocturnal 20 °C, a few nymphs of *A. glycines* developed into adults; however, few offspring were deposited [27]. 

Cultivated soybean, *G. max*, and wild soybean, *Glycine soja* Philipp Franz von Siebold & Joseph Gerhard Zuccarini, 1843, are summer hosts of *A. glycines* [5]. A few studies focused on the characteristics of *A. glycines* fed wild soybeans (*Ag*FW). In summer, *A. glycines* colonize cultivated soybeans; however, a few *A. glycines* are observed on wild soybeans. On the contrary, *A. glycines* colonize wild soybeans in autumn; however, a few *A. glycines* also colonize cultivated soybeans in autumn. Based on the aforementioned phenomena, the occurrence and feeding hosts of *Ag*FW and *A. glycines* fed soybeans (*Ag*FS) differ. The latest research results showed that *Ag*FW was significantly more affected than *Ag*FS by heat waves lasting for 7 d, which reduced the adult reproductive ability and male differentiation proportion in the offspring of *A. glycines* [30]. The phenomena and the research results mentioned above led us to infer that the adaptability of *Ag*FW to temperature and photoperiod is probably different than that of *Ag*FS.

This study investigated the development and reproduction of *Ag*FW in three generations at different temperatures and the morphogenesis at different temperatures and photoperiods, and the data were compared with that of *Ag*FS. *Ag*FW and *Ag*FS were fed on cultivated soybeans and wild soybeans, respectively. They had been reared on a non-original nutritional diet, and the development and reproduction of the aphids were also studied and compared. This information is important for understanding the life cycle of *A. glycines* in Harbin. In regions where *Ag*FW occurs, critical questions regarding soybean production remain unanswered. For example, whether *Ag*FW reproduction results in more gynoparae and males than *Ag*FS reproduction. The results of the morph differentiation in *Ag*FW are important for formulating integrated pest management strategies for *A. glycines* in regions where *G. soja* is common.

## 2. Materials and Methods

### 2.1. Aphid and Hosts

Apterous *Ag*FS were collected at a cultivated soybean field in Northeast Agricultural University, Harbin (126.72° E, 45.74° N), northeast China, in July 2018. Apterous *Ag*FW were collected in September 2018 from an area where wild soybeans grow on Sun Island (126.58° E, 45.79° N), Harbin, where no cultivated soybeans were planted. The population of *Ag*FS was maintained on cultivated soybean seedlings (variety Heinong 51). The population of *Ag*FW was maintained on wild soybean plants, which were both asexually reared in growth chambers at 25 ± 1 °C, 70 ± 5% relative humidity (RH), and a photoperiod of 14L:10D-h with artificial light of 12,000 lux. To maintain *Ag*FS and *Ag*FW, 20–30 aphids each were transferred from mature, heavily infested plants to young, non-infested plants every 2 weeks.

Cultivated soybean seeds (Heinong 51) were purchased from Fangyuan Agricultural Co., Ltd., Wuchang, Heilongjiang Province, China. The cultivated soybean seedlings were planted in circular plastic pots (d × h: 10 × 10 cm). Six to eight cultivated soybean seeds were planted in each pot and placed in a growth chamber. The conditions in the growth chamber were set at 25 ± 1 °C, 70 ± 5% RH, and 14L:10D-h photoperiod with artificial light of 12,000 lux. Cultivated soybean seedlings of 15–20 cm (V2–V3 developmental stage) were used in the experiment. Wild soybean seeds were collected from a wild soybean-growing area on Sun Island. Wild soybeans were planted in plastic pots in a chamber under the same conditions. The seed coat of wild soybeans is thick and hard, which is not conducive to germination. To increase the emergence, we removed parts of the thick testa of the seeds using a knife before planting. Wild soybeans were used for the experiments after 27 days of growth (at florescence) and sprouting.

### 2.2. Development and Reproduction of AgFS and AgFW in Three Generations

Fifty apterous *Ag*FS and *Ag*FW adults were placed in five pots (10 aphids per pot) of soybeans and wild soybeans, respectively. The plants were placed in a growth chamber at 25 ± 1 °C and 70 ± 5% RH, and a 14L:10D-h photoperiod with artificial light of 12,000 lux for a 24 h reproductive period; thereafter, all adults were removed. Newly deposited nymphs (recorded as G_1_) were removed from plants using a small brush. *Ag*FS and *Ag*FW nymphs were transferred to cultivated soybeans and wild soybeans, respectively, and reared individually using the moisturizing cotton method. In brief, a piece of square moisturizing cotton (Shenzhen Xide E-commerce Co., Ltd, Shenzhen, China) (facial cleansing cotton; l × w × h: 2 × 2 × 0.4 cm) was placed on the bottom of a 45 mL, 4 × 4.5 cm (d × h) glass beaker, and a piece of round filter paper with a 4.2 cm diameter was placed on the surface of the moisturizing cotton. The filter paper was cut slightly larger to fix it firmly within the beaker. The filter paper and moisturizing cotton were then wetted with 2200 μL of water by dropping water onto the surface of the filter paper with a pipette. Detached leaves of *G. max* and *G. soja* were cut into 1.5 cm^2^ square pieces using scissors. Each nymph was placed on the reverse side of a square leaf adhered to a filter paper surface. A small drop of water was placed on the surface of the filter paper, and a square piece of leaf was placed on the surface. Due to the application of slight pressure using a small brush, the leaf adhered to the filter paper because of the surface tension of the water. Water was added to the surface of the filter paper every 7–10 d (400 μL each time). The leaves were replaced every 5–7 d or when they became yellowish [27,31].

When G_1_ developed into adults, newly deposited nymphs (G_2_) were placed and reared on soybeans and wild soybeans. When G_2_ developed into adults, newly deposited nymphs (G_3_) were reared on each host. The G_1_, G_2_, and G_3_ groups were reared individually using the moisturizing cotton method. *Ag*FS and *Ag*FW were reared on cultivated soybeans and wild soybeans, respectively. Nymphs were placed in growth chambers at 20, 23, 26, and 29 ± 1 °C and 70 ± 5% RH, and the photoperiod was 14L:10D-h with artificial light of 12,000 lux. Each aphid was considered an experimental unit, and 50 *Ag*FS and *Ag*FW nymphs were used for each temperature treatment. Individual nymphs were examined daily for exuviation and survival. Once they developed into adults, the nymphs deposited by each female were counted and removed daily. The adult lifespan was recorded daily until the death of each adult [28].

### 2.3. Development and Reproduction of AgFS on Wild Soybean and AgFW on Soybean

To investigate the differences in the development and reproduction of *Ag*FS and *Ag*FW on a non-original nutritional diet, we changed the feed: *Ag*FS was fed wild soybeans, and *Ag*FW was fed cultivated soybeans. Each aphid was considered an experimental unit, and 50 newly deposited *Ag*FS and *Ag*FW nymphs were used for each temperature treatment. The nymphs were individually reared using the moisturizing cotton method and placed in growth chambers at 17, 20, 23, 26, 29, or 32 ± 1 °C, 70 ± 5% RH, with a 14L:10D-h photoperiod. Individual nymphs were examined daily for exuviation and survival. Once they developed into adults, the nymphs deposited by each female were counted and removed daily. The adult lifespan was recorded daily until the death of each adult [28].

### 2.4. Morph Differentiation of AgFS and AgFW at Different Temperatures and Photoperiods

The temperature and photoperiod combination treatments were set as follows: (1) four temperatures of 17, 20, 23, and 26 ± 1 °C, with a photoperiod of 10L:14D h and 70 ± 5% RH; and (2) three photoperiods of 8:16, 12:12, and 16:8 (L:D) h, with 17 ± 1 °C and 70 ± 5% RH. For each treatment, 20 apterous adults of *Ag*FS and 50 apterous adults of *Ag*FW (recorded as F_0_) were first reared at 25 ± 1 °C and 70 ± 5% RH with a 14L:10D h photoperiod. Under these conditions, the adult fecundity of *Ag*FS per day was approximately 5–6 offspring per female, and that of *Ag*FW was approximately 2–3 offspring per female. Thus, the number of offspring deposited by 20 *Ag*FS adults per day was similar to that of 50 *Ag*FW adults. After 24 h, the adults were removed, and newly deposited nymphs (recorded as F_1_) were reared in each treatment. When F_1_ developed into adults, the nymphs deposited on days 1, 6, 11, 16, and 21 (F_2_) were reared under the same conditions as aforementioned [26]. All nymphs and adults were reared individually using the moisturizing cotton method. *Ag*FS were reared with cultivated soybeans, and *Ag*FW were reared with wild soybeans. When the F_2_ developed into adults, they were identified as virginoparae, gynoparae, and males based on their morphological characteristics [11]. The proportions of the different morphs that occurred on days 1, 6, 11, 16, and 21 were recorded. Three replicates were performed for each group.

### 2.5. Data Analyses

The nymph stage duration, adult lifespan, and adult fecundity of *Ag*FS and *Ag*FW were calculated according to Chi and Liu 1985 and Chi 1988 [32,33] using TWOSEX-MSChart software (Version number, 2019.06.07) [34]. The intrinsic rate of increase in *Ag*FS and *Ag*FW were calculated by bootstrap technology using the TWOSEX-MSChart [35]. Bootstrap technology with 100,000 resamplings were used to estimate the variances and standard errors [36]. When *Ag*FS was fed cultivated soybean and *Ag*FW was fed wild soybean for three generations, after which the feeds were switched for one generation, the differences in nymph stage duration, adult lifespan, adult fecundity, and intrinsic rate of increase between *Ag*FS and *Ag*FW at each generation and temperature, and the differences in the parameters between *Ag*FS and *Ag*FW at each temperature, were analyzed by a paired bootstrap test (TWOSEX-MSChart) [37].

The percentages of virginoparae, gynoparae, and males in *Ag*FS and *Ag*FW were determined and transformed to arcsine square-root values to fit the normal distribution. Transformed values were used for data analysis. The differences in the percentage of each morph deposited in F_2_ on days 1, 6, 11, 16, and 21 between *Ag*FS and *Ag*FW at each temperature and photoperiod treatment were analyzed using Student’s *t* test (SAS 8.1).

## 3. Results

### 3.1. Development and Reproduction of AgFS and AgFW

**Figure 1:**
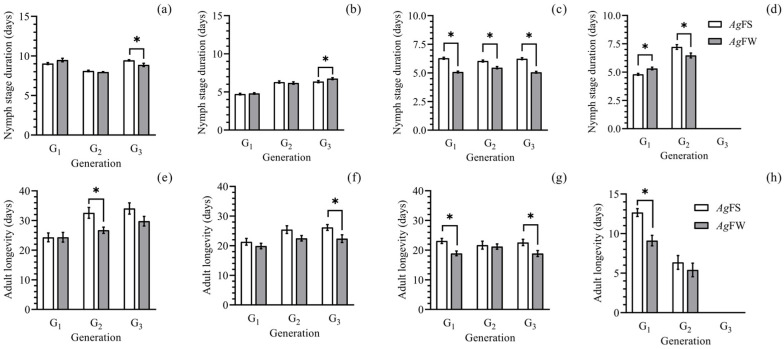
Nymph stage duration and adult longevity of *Ag*FS on soybean and *Ag*FW on wild soybean at different generations. (**a**) Nymph stage duration of *Ag*FS and *Ag*FW at 20 °C. (**b**) Nymph stage duration of *Ag*FS and *Ag*FW at 23 °C. (**c**) Nymph stage duration of *Ag*FS and *Ag*FW at 26 °C. (**d**) Nymph stage duration of *Ag*FS and *Ag*FW at 29 °C. (**e**) Adult longevity of *Ag*FS and *Ag*FW at 20 °C. (**f**) Adult longevity of *Ag*FS and *Ag*FW at 23 °C. (**g**) Adult longevity of *Ag*FS and *Ag*FW at 26 °C. (**h**) Adult longevity of *Ag*FS and *Ag*FW at 29 °C. The data are shown as the mean ± SE. The differences in nymph stage duration or adult longevity between *Ag*FS and *Ag*FW at each generation are marked with ‘*’ (paired bootstrap test, *p* < 0.05).

At 20 °C and 26 °C, the nymph stage durations of the first, second, and third generation *Ag*FW were shorter than or equal to those of *Ag*FS (Figure 1a,c). At 23 °C, the nymph stage duration of the first, second, and third generation *Ag*FW was longer than or equal to that of *Ag*FS (Figure 1b). At 20–26 °C, the adult lifespan of the first, second, and third generation *Ag*FW was shorter than or equal to that of *Ag*FS (Figure 1e–g). At 29 °C, the adult lifespan of the first generation *Ag*FW was shorter than that of *Ag*FS. The adult lifespan of the second generation of *Ag*FW was as long as that of *Ag*FS (Figure 1h).

**Figure 2:**
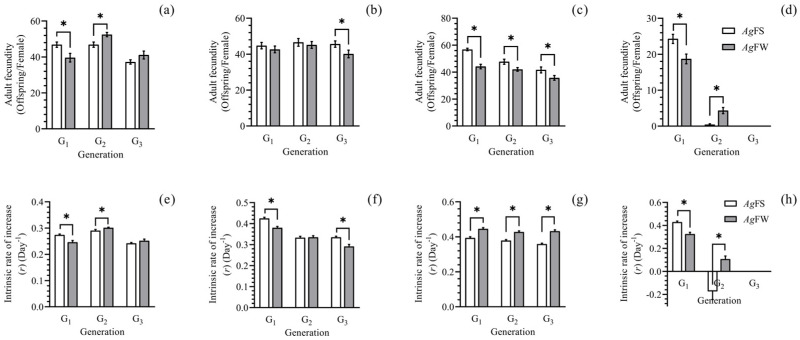
Adult fecundity and intrinsic rate of increase of *Ag*FS on soybean and *Ag*FW on wild soybean at different generations. (**a**) Adult fecundity of *Ag*FS and *Ag*FW at 20 °C. (**b**) Adult fecundity of *Ag*FS and *Ag*FW at 23 °C. (**c**) Adult fecundity of *Ag*FS and *Ag*FW at 26 °C. (**d**) Adult fecundity of *Ag*FS and *Ag*FW at 29 °C. (**e**) Intrinsic rate of increase of *Ag*FS and *Ag*FW at 20 °C. (**f**) Intrinsic rate of increase of *Ag*FS and *Ag*FW at 23 °C. (**g**) Intrinsic rate of increase of *Ag*FS and *Ag*FW at 26 °C. (**h**) Intrinsic rate of increase of *Ag*FS and *Ag*FW at 29 °C. The data are shown as the mean ± SE. The differences in adult fecundity and intrinsic rate of increase between *Ag*FS and *Ag*FW at each generation are marked with ‘*’ (paired bootstrap test, *p* < 0.05).

At 20–29 °C, significant differences existed in adult fecundity and intrinsic rate of increase between *Ag*FW and *Ag*FS of the first, second, and third generation (Figure 2c,g) or certain generations (Figure 2a,b,d,e,f,h). In addition, there were significant differences in biological parameters among different treatments under different temperatures, generations, or populations (Tables S1 and S2).

To investigate the performance of *Ag*FW on the non-origin host, *Ag*FW were reared on cultivated soybeans. At 17 °C to 32 °C, *Ag*FS and *Ag*FW developed and reproduced successfully on wild and cultivated soybean; however, certain significant differences existed in the nymph stage duration, adult lifespan (Table 1 and Table S3), adult fecundity, and intrinsic rate of increase between *Ag*FS and *Ag*FW (Table 2 and Table S4). At 17 °C, *Ag*FW fed on soybean had a longer nymph stage duration and adult lifespan (Table 1).

### 3.2. Morph Differentiation of AgFS and AgFW at Different Temperatures and Photoperiods

**Figure 3:**
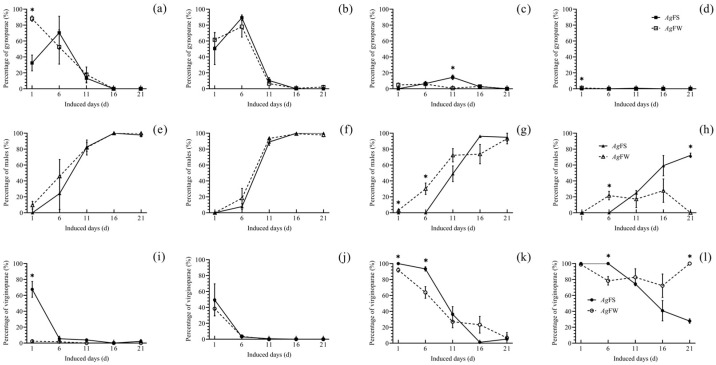
Percentage of different *A. glycines* morphs deposited in the F_2_ when the F_1_ of *Ag*FS and *Ag*FW was exposed to different temperatures with a photoperiod of 10L:14D. (**a**) Gynoparae percentage of *Ag*FS and *Ag*FW at 17 °C. (**b**) Gynoparae percentage of *Ag*FS and *Ag*FW at 20 °C. (**c**) Gynoparae percentage of *Ag*FS and *Ag*FW at 23 °C. (**d**) Gynoparae percentage of *Ag*FS and *Ag*FW at 26 °C. (**e**) Male percentage of *Ag*FS and *Ag*FW at 17 °C. (**f**) Male percentage of *Ag*FS and *Ag*FW at 20 °C. (**g**) Male percentage of *Ag*FS and *Ag*FW at 23 °C. (**h**) Male percentage of *Ag*FS and *Ag*FW at 26 °C. (**i**) Virginoparae percentage of *Ag*FS and *Ag*FW at 17 °C. (**j**) Virginoparae percentage of *Ag*FS and *Ag*FW at 20 °C. (**k**) Virginoparae percentage of *Ag*FS and *Ag*FW at 23 °C. (**l**) Virginoparae percentage of *Ag*FS and *Ag*FW at 26 °C. The data are shown as the mean ± SE. The differences in percentage of gynoparae, males, or virginoparae between *Ag*FS and *Ag*FW at days 1, 6, 11, 16, and 21 are marked with ‘*’ (Student’s *t*-test, *p* < 0.05).

At 17 °C and a 10L:14D-h photoperiod, on day 1, a higher percentage of gynoparae (Figure 3a) and a lower percentage of virginoparae of *Ag*FW (Figure 3i) were deposited than that of *Ag*FS. At 20 °C and a 10L:14D-h photoperiod, on days 1, 6, 11, 16, and 21, the percentages of gynoparae (Figure 3b), males (Figure 3f), and virginoparae (Figure 3j) deposited by *Ag*FW were equal to those of *Ag*FS. At 23 °C and a 10L:14D-h photoperiod, on days 1 and 6, males were deposited by *Ag*FW. No males were deposited by *Ag*FS (Figure 3g). On day 11, a smaller percentage of gynoparae of *Ag*FW (0.74 ± 0.74%) was deposited than that of *Ag*FS (14.49 ± 2.84%) (Figure 3c). At 26 °C and a 10L:14D-h photoperiod, *Ag*FW deposited gynoparae on day 1 (Figure 3d) and males on day 6 (Figure 3h). The offspring deposited by *Ag*FS on days 1–6 were virginoparae (Figure 3l). On day 21, the offspring treated with *Ag*FW were all virginoparae (Figure 3l), and the offspring treated with *Ag*FS were male (Figure 3h) and virginoparae (Figure 3l).

**Figure 4:**
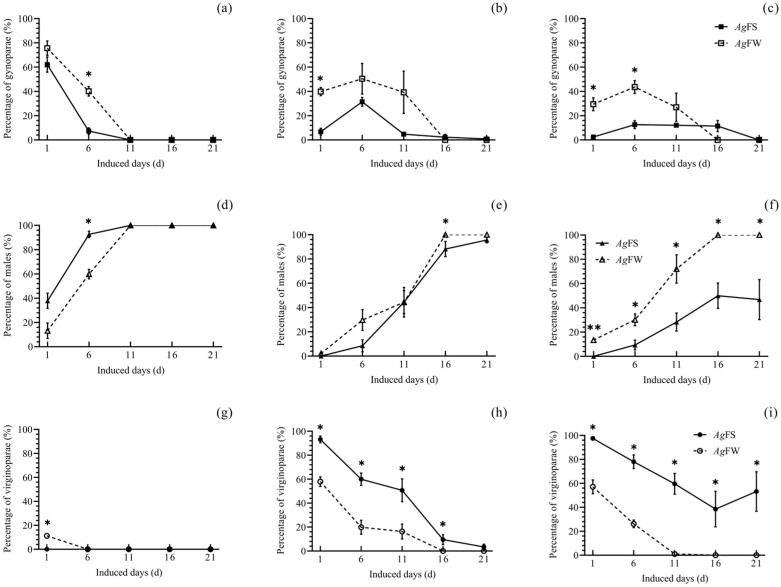
Percentage of different *A. glycines* morphs induced in the F_2_ when the F_1_ of *Ag*FS and *Ag*FW was exposed to different photoperiods and 17 °C. (**a**) Gynoparae percentage of *Ag*FS and *Ag*FW at an 8L:16D-h photoperiod. (**b**) Gynoparae percentage of *Ag*FS and *Ag*FW at a 12L:12D-h photoperiod. (**c**) Gynoparae percentage of *Ag*FS and *Ag*FW at a 16L:8D-h photoperiod. (**d**) Male percentage of *Ag*FS and *Ag*FW at an 8L:16D-h photoperiod. (**e**) Male percentage of *Ag*FS and *Ag*FW at a 12L:12D-h photoperiod. (**f**) Male percentage of *Ag*FS and *Ag*FW at a 16L:8D-h photoperiod. (**g**) Virginoparae percentage of *Ag*FS and *Ag*FW at an 8L:16D-h photoperiod. (**h**) Virginoparae percentage of *Ag*FS and *Ag*FW at a 12L:12D-h photoperiod. (**i**) Virginoparae percentage of *Ag*FS and *Ag*FW at a 16L:8D-h photoperiod. The data are shown as the mean ± SE. The differences in percentage of gynoparae, males, or virginoparae between *Ag*FS and *Ag*FW are marked with ‘*’ (Student’s *t*-test, *p* < 0.05).

At 17 °C and an 8L:16D-h photoperiod, on day 6, a higher percentage of gynoparae (Figure 4a) and lower percentage of males (Figure 4d) were deposited by *Ag*FW than by *Ag*FS. At 17 °C and a 12L:12D-h photoperiod, on days 1 and 16, a higher percentage of gynoparae (Figure 4b) and males (Figure 4e) were deposited by *Ag*FW than that by *Ag*FS. A lower percentage of virginoparae of *Ag*FW was deposited on days 1–16 than that of *Ag*FS (Figure 4h). At 17 °C and a 16L:8D-h photoperiod, on days 1 and 6, the percentages of *Ag*FW gynoparae were higher than those of *Ag*FS (Figure 4c). On days 1 to 21, a higher percentage of males (Figure 4f) and a lower percentage of virginoparae (Figure 4i) were deposited by *Ag*FW than by *Ag*FS. In addition, there were significant differences in percentage of gynoparae or male of *Ag*FW and *Ag*FS at each temperatures or photoperiods (Tables S5 and S6).

## 4. Discussion

Previous studies proved that aphids could have different host-specialized biotypes [38]. In *A. gossypii*, non-original hosts led to aphid death and even population collapse [39,40]. However, these phenomena were not observed in *A. glycines*. When *Ag*FS and *Ag*FW were transferred to rearing on the non-original host, both developed into adults and were successfully reproduced (Table 1 and Table 2). Therefore, we concluded that *Ag*FW has probably not evolved a wild soybean-specialized biotype in soybean aphids. However, more evidence for the identification of a wild soybean-specialized biotype should be conducted in other natural populations of *A. glycines* collected from wild soybean. 

This study is the first to report on *Ag*FW regarding the temperature and photoperiod set in trials. The development, reproduction, and morphogenesis of *Ag*FS have been studied in previous studies, and the data are comparable. *Ag*FS can survive and maintain a population on cultivated soybean at a temperature range from 20 to 29 °C (Figure 1 and Figure 2), which is consistent with studies focused on the effect of temperature on a single generation of *A. glycines* [25,41,42]. At 29 °C, the nymph stage duration and adult longevity of *Ag*FS (Figure 1) were similar to those of another study [42]. At 20 °C, the adult fecundity of the first-generation *Ag*FS (Figure 2) was lower than the reported result, 63.5 ± 2.2 offspring per female [25]; however, it was higher than the reported 27.87 ± 15.64 offspring per female [41]. The performance of *A. glycines* in development and reproduction was affected by the rearing method [31] and the different populations of *A. glycines* used in other studies [43]. In our study, *A. glycines* were collected from Harbin, China, and reared using the moisturizing cotton method. The differences in the adult fecundity of *A. glycines* among the studies could be partially attributed to the different rearing methods and origins of the *A. glycines* used. At low temperatures and short photoperiods, gynoparae and males of *Ag*FS and *Ag*FW were deposited (Figure 3 and Figure 4), which is consistent with the literature on *A. glycines* fed on cultivated soybeans [26,44,45].

At a 10L:14D h photoperiod and 23 °C, males were deposited by *Ag*FW on days 1–6. However, no gynoparae (the morph producing oviparae) were deposited (Figure 3). This phenomenon is probably protective in *Ag*FW. This result is inconsistent with the reported life cycle of *A. glycines*, in which gynoparae occur earlier than males and migrate to winter hosts to produce oviparae [2]. The males deposited by *Ag*FW might act as explorers in searching for winter hosts in the life cycle of *A. glycines*, which occurs earlier in the field, and then migrates to winter hosts where they wait for oviparae. When the oviparae develop into adults, the males can mate with them. If males of *A. glycines* occur later than oviparae in nature, oviparae must wait for males on the winter hosts, which would cause a high risk of pre-reproductive death and hinder population breeding. Thus, the *Ag*FW is probably an important component of the *A. glycines* life cycle, which has been neglected until now. A low temperature and short photoperiod combination is commonly observed in September in Harbin. Therefore, the results of the morph differentiation of *Ag*FW at 23 °C and 10L:14D-h can reflect what would be observed in nature to a certain extent. Global warming is an indisputable phenomenon. Heilongjiang Province in northeast China is one of the regions experiencing a rapid temperature increase [46]. In regions with rising environmental temperatures, native organisms are experiencing increased survival pressures. As global warming intensifies in Harbin, Heilongjiang Province, temperatures of 26 °C in autumn with a short photoperiod might occur. In autumn at 26 °C and a 10L:14D h photoperiod, males and gynoparae probably will be deposited by *Ag*FW earlier in the field than by *Ag*FS, as was presented in the laboratory (Figure 3). With its reproductive adaptability strategy, *A. glycines* is likely to continue to flourish in Harbin, northeast China, where the local environmental temperature is increasing.

Wild soybeans are regarded as important farmland weeds when grown in large numbers in soybean fields, which would result in reduced production [47,48]. Our study showed that the males and gynoparae of *Ag*FW are deposited earlier than usual or in large proportions, which helps *A. glycines* to complete its life cycle. The larger autumn populations of *A. glycines* would probably lead to an excessive summer population that would damage cultivated soybean in the following year. Thus, measures to eradicate wild soybeans around cultivated soybean is important for the integrated management of *A. glycines* in the field, which would probably reduce the numbers of *A. glycines* gynoparae and males occurring in autumn to some extend. However, our study had some limitations in exploring the adaptability of the different populations of *Ag*FW, which were collected from different wild soybeans in nature. More populations of *Ag*FW would improve our understanding of *A. glycines* fed on wild soybean. Be that as it may, our work provides an important understanding of the adaptability of *A. glycines* to temperature and photoperiod in the laboratory, perfecting the life cycle of *A. glycines* in Harbin, northeast China.

## 5. Conclusions

Our study confirmed that the temperature adaptability between *Ag*FS and *Ag*FW was significantly different under multiple generations. We further demonstrated that the morph differentiation of *Ag*FW performed differently from *Ag*FS in various temperature and photoperiod conditions. These results suggested that *Ag*FW had significant differences in morph differentiation, temperature adaptability, and photoperiod adaptability, compared with *Ag*FS. It is crucial to understand the life cycle of *A. glycines* fed on different hosts in Harbin, northeast China, and provide a sound foundation for developing insect control strategies.

## Figures and Tables

**Table 1:** Nymph stage duration and adult lifespan of *Ag*FS on wild soybean and *Ag*FW on soybean.

Temperature (°C)	Nymph Stage Duration (Day)		Adult Lifespan (Day)	
	*Ag*FS Fed on Wild Soybean	*Ag*FW Fed on Soybean	*Ag*FS Fed on Wild Soybean	*Ag*FW Fed on Soybean
17	10.06 ± 0.09 a	11.20 ± 0.11 a *	28.22 ± 1.93 ab	38.70 ± 2.18 a *
20	7.06 ± 0.10 b	8.15 ± 0.11 b *	30.47 ± 1.72 a	39.85 ± 1.46 a *
23	6.57 ± 0.12 c	6.24 ± 0.09 c *	24.63 ± 1.68 b	24.18 ± 0.99 b
26	5.54 ± 0.09 d	6.12 ± 0.14 c *	20.50 ± 1.27 c	23.78 ± 0.79 b *
29	4.60 ± 0.09 e	5.22 ± 0.09 d *	8.51 ± 1.03 d	16.38 ± 0.83 c *
32	5.24 ± 0.11 f	5.11 ± 0.07 d	9.10 ± 0.87 d	10.81 ± 0.60 d

Note: Data are shown as mean ± SE. The differences in nymph stage duration or adult lifespan of each population among different temperatures are marked with a lowercase letter behind the mean ± SE. The differences in nymph stage duration and adult lifespan between AgFS and AgFW at each temperature are marked with ‘*’ (paired bootstrap test, *p* < 0.05).

**Table 2:** Adult fecundity and intrinsic rate of increase of *Ag*FS on wild soybean and *Ag*FW on soybean.

Temperature (°C)	Adult Fecundity (Offspring/Female)		Intrinsic Rate of Increase (Day^−1^)	
	*Ag*FS Fed on Wild Soybean	*Ag*FW Fed on Soybean	*Ag*FS Fed on Wild Soybean	*Ag*FW Fed on Soybean
17	42.30 ± 2.10 c	44.20 ± 2.02 b	0.2321 ± 0.0024 c	0.1989 ± 0.0019 d *
20	51.32 ± 2.01 a	45.28 ± 1.18 b *	0.3236 ± 0.0059 b	0.2772 ± 0.0047 c *
23	44.80 ± 2.18 bc	45.12 ± 1.77 ab	0.3375 ± 0.0078 b	0.3592 ± 0.0049 b *
26	48.06 ± 1.91 ab	49.22 ± 1.42 a	0.4182 ± 0.0066 a	0.3743 ± 0.0071 ab *
29	14.96 ± 1.56 d	32.22 ± 1.99 c *	0.3459 ± 0.0126 b	0.3884 ± 0.0099 a *
32	6.12 ± 0.62 e	9.08 ± 0.80 d *	0.1983 ± 0.0150 d	0.2579 ± 0.0165 c *

Note: Data are shown as mean ± SE. The differences in adult fecundity or intrinsic rate of increase of each population among different temperatures are marked with a lowercase letter behind the mean ± SE. The differences in adult fecundity or intrinsic rate of increase between *Ag*FS and *Ag*FW at each temperature are marked with ‘*’ (paired bootstrap test, *p* < 0.05).

## Data Availability

Data will be made available on request.

## Supplementary Materials

The following supporting information can be downloaded at: https://www.mdpi.com/article/10.3390/insects15100816/s1, Table S1: Under different analysis modes, the nymph stage duration and adult longevity of *Ag*FS on soybean and *Ag*FW on wild soybean at different generations; Table S2: Under different analysis modes, the adult fecundity and intrinsic rate of increase of *Ag*FS on soybean and *Ag*FW on wild soybean at different generations; Table S3: Under different analysis modes, nymph stage duration and adult lifespan of *Ag*FS on wild soybean and *Ag*FW on soybean;.Table S4: Under different analysis modes, adult fecundity and intrinsic rate of increase of *Ag*FS on wild soybean and *Ag*FW on soybean; Table S5: The difference analysis of the percentage of gynoparae of *A. glycines* at each temperatures; Table S6: The difference analysis of the percentage of gynoparae of *A. glycines* at each photoperiod.
