# Supplementary material for: Adaptability of the Soybean Aphid Aphis glycines (Hemiptera: Aphididae) to Temperature and Photoperiod in a Laboratory Experiment
Source: Insects. 2024 Oct 17;15(10):816. doi: 10.3390/insects15100816 (PMC11508913; doi:10.3390/insects15100816)
Supplement: Supplementary file 1 [file insects-15-00816-s001.zip › Supplementary information/Table S1.pdf]

Table S1. Under different analysis modes, the nymph stage duration and adult longevity of *Ag*FS on soybean and *Ag*FW on wild soybean at different generations.

Tem. (°C)	Nymph stage duration (day)						Adult lifespan (day)					
	***Ag*FS**			***Ag*FW**			***Ag*FS**			***Ag*FW**		
	**G_1**	**G_2**	**G_3**	**G_1**	**G_2**	**G_3**	**G_1**	**G_2**	**G_3**	**G_1**	**G_2**	**G_3**
20	9.04±0.12<br>bc (bc)<br>a (b)	8.10±0.10<br>d (d)<br>a (c)	9.44±0.08<br>a (a)<br>a (a)	9.48±0.23<br>ab (ab)<br>a (a)	7.96±0.06<br>d (d)<br>a (c)	8.87±0.21<br>c (c)<br>b (b)	24.32±1.50<br>cde (c)<br>a (bcd)	32.58±1.81<br>a (a)<br>a (a)	34.06±1.89<br>a (a)<br>a (a)	24.35±1.65<br>cde (c)<br>a (bc)	26.72±1.07<br>bc (bc)<br>b (ab)	29.78±1.62<br>ab (ab)<br>ab (a)
23	4.72±0.10<br>j (c)<br>d (f)	6.29±0.14<br>g (b)<br>c (e)	6.35±0.14<br>g (b)<br>d (e)	4.80±0.11<br>j (c)<br>d (h)	6.17±0.16<br>g (b)<br>c (e)	6.74±0.14<br>ef (a)<br>c (d)	21.32±1.13<br>efg (cd)<br>ab (d)	25.44±1.30<br>cd (ab)<br>bc (bc)	26.16±0.99<br>bc (a)<br>b (b)	19.90±0.94<br>fg (d)<br>b (de)	22.5±0.91<br>de (bc)<br>cd (c)	22.37±1.30<br>def (bcd)<br>c (cd)
26	6.28±0.09<br>g (a)<br>b (e)	6.04±0.09<br>g (a)<br>c (e)	6.24±0.09<br>g (a)<br>d (e)	5.08±0.08<br>i (c)<br>c (g)	5.46±0.1<br>h (b)<br>d (f)	5.06±0.08<br>i (c)<br>e (g)	23.10±0.86<br>de (a)<br>a (cd)	21.66±1.34<br>efg (ab)<br>d (d)	22.58±1.06<br>def (a)<br>c (cd)	18.90±0.80<br>g (b)<br>b (e)	21.18±0.89<br>efg (ab)<br>d (cde)	18.86±1.00<br>g (b)<br>d (e)
29	4.80±0.09<br>j (d)<br>d (f)	7.22±0.21<br>e (a)<br>b (d)	-	5.32±0.12<br>hi (c)<br>c (fg)	6.47±0.22<br>fg (b)<br>c (de)	-	12.66±0.50<br>g (a)<br>c (e)	6.35±0.87<br>j (c)<br>e (f)	-	9.11±0.66<br>h (b)<br>d (f)	5.40±0.86<br>j (c)<br>e (g)	-

Note: These Data were same as Figure 1. Data are shown as mean ± SE. The differences in adult nymph stage duration or adult lifespan of *Ag*FS and *Ag*FW (all data, 22 groups) were marked with first lowercase letter. The differences in nymph stage duration or adult lifespan of *Ag*FS and *Ag*FW at same temperature (12 groups for 20, 23, and 26 °C, 8 groups for 29 °C) were marked with second lowercase letter. The differences in nymph stage duration or adult lifespan of *Ag*FS and *Ag*FW at same generation (8 groups for G_1 and G_2, 6 groups for G_3) were marked with lowercase letter. The differences in nymph stage duration or adult lifespan at same population of *Ag*FS or *Ag*FW (11 groups for each population)were marked with forth lowercase letter (paired bootstrap test, $P < 0.05$).
