# Supplementary material for: Adaptability of the Soybean Aphid Aphis glycines (Hemiptera: Aphididae) to Temperature and Photoperiod in a Laboratory Experiment
Source: Insects. 2024 Oct 17;15(10):816. doi: 10.3390/insects15100816 (PMC11508913; doi:10.3390/insects15100816)
Supplement: Supplementary file 1 [file insects-15-00816-s001.zip › Supplementary information/Table S2.pdf]

Table S2. Under different analysis modes, the adult fecundity and intrinsic rate of increase of *Ag*FS on soybean and *Ag*FW on wild soybean at different generations.

Tem. (°C)	Adult fecundity (offspring/ female)	Intrinsic rate of increase (Day^{-1})

Note: These Data were same as Figure 2. Data are shown as mean ± SE. The differences in adult fecundity or intrinsic rate of increase of *Ag*FS and *Ag*FW (all data, 22 groups) were marked with first lowercase letter. The differences in adult fecundity or intrinsic rate of increase of AgFS and AgFW at same temperature (12 groups for 20, 23, and 26 °C, 8 groups for 29 °C) were marked with second lowercase letter. The differences in adult fecundity or intrinsic rate of increase of *Ag*FS and *Ag*FW at same generation (8 groups for G_1 and G_2, 6 groups for G_3) were marked with third lowercase letter. The differences in adult fecundity or intrinsic rate of increase at same population of *Ag*FS or *Ag*FW (11 groups for each population) were marked with forth lowercase letter (paired bootstrap test, $P < 0.05$).

	***Ag*FS**			***Ag*FW**			***Ag*FS**			***Ag*FW**		
	**G_1**	**G_2**	**G_3**	**G_1**	**G_2**	**G_3**	**G_1**	**G_2**	**G_3**	**G_1**	**G_2**	**G_3**
	46.96±1.49	46.86±1.46	37.22±1.20	39.61±2.42	52.46±1.20	41.09±2.19	0.274±0.0035	0.2905±0.0043	0.2419±0.0032	0.2460±0.0067	0.3011±0.0022	0.2518±0.0062
20	c (b)	c (b)	fg (c)	efg (c)	b (a)	defg (c)	h (c)	g (b)	i (d)	i (d)	f (a)	i (d)
	b (b)	b (b)	bc (d)	c (bc)	a (a)	abc (b)	e (f)	e (e)	e (g)	f (f)	d (e)	e (f)
	44.74±1.77	46.56±2.16	45.61±1.76	42.63±1.87	45.11±1.91	40.11±2.13	0.4251±0.0052	0.3331±0.0065	0.3352±0.0051	0.3804±0.0064	0.3350±0.0077	0.2916±0.0096
23	cde (ab)	cd (a)	cd (a)	cde (ab)	cde (ab)	efg (b)	b (a)	e (c)	e (c)	c (b)	e (c)	fgh (d)
	bc (bc)	bc (b)	a (bc)	bc (b)	bc (b)	bc (bc)	b (a)	c (d)	c (d)	c (c)	c (d)	d (e)
	56.78±0.91	47.72±1.88	41.62±2.19	44.14±1.67	42.02±1.34	35.72±1.74	0.3939±0.0066	0.3787±0.0056	0.3587±0.0047	0.4456±0.0068	0.4276±0.0061	0.4320±0.0079
26	a (a)	c (b)	def (c)	cde (bc)	de (c)	g (d)	c (c)	c (c)	d (d)	a (a)	b (b)	ab (ab)
	a (a)	b (b)	ab (cd)	bc (b)	c (b)	c (c)	cf (b)	b (b)	b (c)	a (a)	a (b)	a (ab)
	24.28±1.26	0.43±0.23		18.71±1.35	4.33±0.856		0.4289±0.0091	-0.1728±0.0724		0.3247±0.015	0.1077±0.0260	
29	h (a)	k (d)	-	i (b)	j (c)	-	ab (a)	k (d)	-	ef (b)	j (c)	-
	d (e)	e (f)		e (d)	d (e)		ab (a)	g (h)		d (de)	f (g)	
