# Supplementary material for: Adaptability of the Soybean Aphid Aphis glycines (Hemiptera: Aphididae) to Temperature and Photoperiod in a Laboratory Experiment
Source: Insects. 2024 Oct 17;15(10):816. doi: 10.3390/insects15100816 (PMC11508913; doi:10.3390/insects15100816)
Supplement: Supplementary file 1 [file insects-15-00816-s001.zip › Supplementary information/Table S3.pdf]

Table S3. Under different analysis modes, nymph stage duration and adult lifespan of *Ag*FS on wild soybean and *Ag*FW on soybean.

**Temperature (°C)**	**Nymph stage duration (day)**		**Adult lifespan (day)**	
	***Ag*FS fed on wild soybean**	***Ag*FW fed on soybean**	***Ag*FS fed on wild soybean**	***Ag*FW fed on soybean**
17	10.06±0.09 b	11.20±0.11 a	28.22±1.93 bc	38.70±2.18 a
20	7.06±0.10 d	8.15±0.11 c	30.47±1.72 b	39.85±1.46 a
23	6.57±0.12 e	6.24±0.09 f	24.63±1.68 cd	24.18±0.99 cd
26	5.54±0.09 g	6.12±0.14 f	20.50±1.27 e	23.78±0.79 d
29	4.60±0.09 i	5.22±0.09 h	8.51±1.03 g	16.38±0.83 f
32	5.24±0.11 h	5.11±0.07 h	9.10±0.87 g	10.81±0.60 g

Note: These Data were same as Table 1. Data are shown as mean ± SE. The differences in nymph stage duration or adult lifespan among all treatments (all data, 12 groups) are marked with a lowercase letter (paired bootstrap test, $P < 0.05$).
