# Supplementary material for: Adaptability of the Soybean Aphid Aphis glycines (Hemiptera: Aphididae) to Temperature and Photoperiod in a Laboratory Experiment
Source: Insects. 2024 Oct 17;15(10):816. doi: 10.3390/insects15100816 (PMC11508913; doi:10.3390/insects15100816)
Supplement: Supplementary file 1 [file insects-15-00816-s001.zip › Supplementary information/Table S4.pdf]

Table S4. Under different analysis modes, adult fecundity and intrinsic rate of increase of *Ag*FS on wild soybean and *Ag*FW on soybean.

Temperature (°C)	Adult fecundity (offspring/female)		Intrinsic rate of increase (Day^{-1})	
	***Ag*FS fed on wild soybean**	***Ag*FW fed on soybean**	***Ag*FS fed on wild soybean**	***Ag*FW fed on soybean**
17	42.30±2.10 d	44.20±2.02 cd	0.2321±0.0024 g	0.1989±0.0019 h
20	51.32±2.01 a	45.28±1.18 cb	0.3236±0.0059 e	0.2772±0.0047 f
23	44.80±2.18 bcd	45.12±1.77 bcd	0.3375±0.0078 e	0.3592±0.0049 cd
26	48.06±1.91 abc	49.22±1.42 ab	0.4182±0.0066 a	0.3743±0.0071 e
29	14.96±1.56 df	32.22±1.99 e	0.3459±0.0126 de	0.3884±0.0099 b
32	6.12±0.62 h	9.08±0.80 g	0.1983±0.0150 h	0.2579±0.0165 fg

Note: These Data were same as Table 2. Data are shown as mean ± SE. The differences in nymph stage duration or adult lifespan among all treatments (all data, 12 groups) are marked with a lowercase letter (paired bootstrap test, $P < 0.05$).
