# Supplementary material for: Adaptability of the Soybean Aphid Aphis glycines (Hemiptera: Aphididae) to Temperature and Photoperiod in a Laboratory Experiment
Source: Insects. 2024 Oct 17;15(10):816. doi: 10.3390/insects15100816 (PMC11508913; doi:10.3390/insects15100816)
Supplement: Supplementary file 1 [file insects-15-00816-s001.zip › Supplementary information/Table S5.pdf]

Table S5. The difference analysis of the percentage of gynoparae of *A. glycines* at each temperatures.

**Tem. (°C)**	**Percentage of gynoparae (%)**									
	***Ag*FS**					***Ag*FW**				
	**Day 1**	**Day 6**	**Day 11**	**Day 16**	**Day 21**	**Day 1**	**Day 6**	**Day 11**	**Day 16**	**Day 21**
17	32.49±9.91<br>ab	70.39±20.77<br>a	13.32±5.92<br>b	0±0<br>b	0±0<br>b	88.11±3.37<br>a	52.55±21.54<br>ab	17.99±9.41<br>b	0±0<br>b	0±0<br>b
20	50.53±20.21<br>ab	89.22±3.61<br>a	10.43±3.65<br>b	0±0<br>b	0±0<br>b	61.62±8.73<br>a	78.08±13.14<br>a	6.51±0.29<br>b	0.74 ±0.74<br>b	2.22±2.22<br>b
23	0±0<br>b	6.59±2.74<br>a	14.49±2.84<br>a	2.71±1.11<br>ab	0±0<br>b	4.82±2.49<br>ab	5.97±0.75<br>a	0.74±0.74<br>b	3.06±1.81<br>b	0±0<br>b
26	0±0<br>a	0±0<br>a	0.71±0.71<br>ab	0±0<br>a	0±0<br>a	1.18±0.28<br>a	0±0<br>a	0±0<br>a	0±0<br>a	0±0<br>a

Note: These Data were same as Figure 3. Data are shown as mean ± SE. The differences in percentage of gynoparae of *Ag*FS and *Ag*FW at same temperature (10 groups for each temperature) were marked with lowercase letter (two-way ANOVA and Tukey test, $P < 0.05$).

Tem. (°C)	Percentage of males (%)									
	*Ag*FS					*Ag*FW				
	Day 1	Day 6	Day 11	Day 16	Day 21	Day 1	Day 6	Day 11	Day 16	Day 21
17	0±0<br>c	24.17±20.2<br>c	82.72±5.51<br>ab	100±0<br>a	97.78±2.22<br>a	9.55±4.81<br>c	45.86±21.06<br>bc	82.01±9.41<br>ab	100±0<br>a	100±0<br>a
20	0±0<br>c	7.79±3.64<br>bc	88.98±4.18<br>a	100±0<br>a	100±0<br>a	0±0<br>c	18.37±12.25<br>b	93.49±0.29<br>a	99.26±0.74<br>a	97.78±2.22<br>a
23	0±0<br>d	0±0<br>d	49.04±9.84<br>bc	96.2±1.12<br>a	94.87±5.13<br>a	3.23±0.63<br>cd	30.23±7.20<br>c	72.33±8.39<br>bc	73.73±12.23<br>ab	93.33±6.67<br>a
26	0±0<br>c	0±0<br>c	24.86±0.73<br>ab	59.22±12.8<br>ab	72.22±2.78<br>a	0±0<br>c	21.63±5.36<br>bc	17.22±10.64<br>bc	27.78±14.7<br>abc	0±0<br>c

Note: These Data were same as Figure 3. Data are shown as mean ± SE. The differences in percentage of male of *Ag*FS and *Ag*FW at same temperature (10 groups for each temperature) were marked with lowercase letter (two-way ANOVA and Tukey test, $P < 0.05$).

Tem. (°C)	Percentage of virginoparae (%)									
	*Ag*FS					*Ag*FW				
	**Day 1**	**Day 6**	**Day 11**	**Day 16**	**Day 21**	**Day 1**	**Day 6**	**Day 11**	**Day 16**	**Day 21**
17	67.51±9.91 a	5.44±3.16 b	3.97±0.52 b	0±0 b	2.22±2.22 b	2.34±1.63 b	1.59±1.59 b	0±0 b	0±0 b	0±0 b
20	49.47±20.21 a	2.99±0.69 b	0.59±0.59 b	0±0 b	0±0 b	38.38±8.73 a	3.54±1.78 b	0±0 b	0±0 b	0±0 b
23	100±0 a	93.41±2.74 ab	36.47±9.49 c	1.09±1.09 d	5.13±5.13 d	91.95±2.6 ab	63.8±7.32 b	26.93±7.65 cd	23.2 ±10.51 cd	6.67±6.67 cd
26	100±0 a	100±0 a	74.44±0.02 b	40.78±12.8 b	27.78±2.78 c	98.82±0.28 ab	78.37±5.36 ab	82.78±10.64 ab	72.22±14.7 ab	100±0 a

Note: These Data were same as Figure 3. Data are shown as mean ± SE. The differences in percentage of virginoparae of *Ag*FS and *Ag*FW at same temperature (10 groups for each temperature) were marked with lowercase letter (two-way ANOVA and Tukey test, $P < 0.05$).
