# Supplementary material for: Adaptability of the Soybean Aphid Aphis glycines (Hemiptera: Aphididae) to Temperature and Photoperiod in a Laboratory Experiment
Source: Insects. 2024 Oct 17;15(10):816. doi: 10.3390/insects15100816 (PMC11508913; doi:10.3390/insects15100816)
Supplement: Supplementary file 1 [file insects-15-00816-s001.zip › Supplementary information/Table S6.pdf]

Table S6. The difference analysis of the percentage of gynoparae of *A. glycines* at each photoperiod.

Photoperiod	Percentage of gynoparae (%)									
	*Ag*FS					*Ag*FW				
	Day 1	Day 6	Day 11	Day 16	Day 21	Day 1	Day 6	Day 11	Day 16	Day 21
8L:16D	62.09±6.29 a	7.39±2.62 c	0±0 d	0±0 d	0±0 d	75.63±5.92 a	40.13±3.96 b	0±0 d	0±0 d	0±0 d
12L:12D	6.83±2.89 a	31.48±3.64 a	4.83±1.77 b	2.30±2.30 b	0.90±0.90 b	39.90±3.390 a	50.42±12.63 a	39.30±17.40 a	0±0 b	0±0 b
16L:8D	2.48±1.24 c	12.62±3.28 b	12.15±1.62 bc	11.48±4.64 bc	0±0 c	29.44±5.38 ab	43.64±5.34 a	26.94±11.61 ab	0±0 c	0±0 c

Note: These Data were same as Figure 4. Data are shown as mean ± SE. The differences in percentage of gynoparae of *Ag*FS and *Ag*FW at same temperature (10 groups for each photoperiod) were marked with lowercase letter (two-way ANOVA and Tukey test, $P < 0.05$).

Photoperiod	Percentage of males (%)									
	*Ag*FS					*Ag*FW				
	**Day 1**	**Day 6**	**Day 11**	**Day 16**	**Day 21**	**Day 1**	**Day 6**	**Day 11**	**Day 16**	**Day 21**
8L:16D	37.91±6.29 d	92.61±2.62 b	100±0 a	100±0 a	100±0 a	13.20±6.36 e	59.87±3.96 c	100±0 a	100±0 a	100±0 a
12L:12D	0±0 d	8.54±4.90 cd	44.49±9.44 b	88.18±6.3 a	95.63±2.28 a	2.14±1.11 d	29.72±8.61 bc	44.38±12.19 b	100±0 a	100±0 a
16L:8D	0±0 c	9.34±4.01 c	28.25±7.47 b	49.97±10.47 b	46.79±16.51 b	13.43±0.91 c	30.13±4.83 b	72.05±11.67 b	100±0 a	100±0 a

Note: These Data were same as Figure 4. Data are shown as mean ± SE. The differences in percentage of male of *Ag*FS and *Ag*FW at same temperature (10 groups for each photoperiod) were marked with lowercase letter (two-way ANOVA and Tukey test, $P < 0.05$).

Photoperiod	Percentage of virginoparae (%)									
	*Ag*FS					*Ag*FW				
	**Day 1**	**Day 6**	**Day 11**	**Day 16**	**Day 21**	**Day 1**	**Day 6**	**Day 11**	**Day 16**	**Day 21**
8L:16D	0±0 b	0±0 b	0±0 b	0±0 b	0±0 b	11.17±0.46 a	0±0 b	0±0 b	0±0 b	0±0 b
12L:12D	93.17±2.89 a	59.98±5.21 b	50.68±9.56 b	9.52±4.09 cd	3.47±2.25 cd	57.96±4.00 b	19.85±5.85 c	16.32±6.24 c	0±0 d	0±0 d
16L:8D	97.52±1.24 a	78.04±5.67 ab	59.6±8.68 bc	38.55±14.84 bc	53.21±16.51 bc	57.13±5.72 bc	26.23±3.19 c	1.01±1.01 d	0±0 d	0±0 d

Note: These Data were same as Figure 4. Data are shown as mean ± SE. The differences in percentage of virginoparae of *Ag*FS and *Ag*FW at same temperature (10 groups for each photoperiod) were marked with lowercase letter (two-way ANOVA and Tukey test, $P < 0.05$).
